# The Association between Major Adverse Cardiovascular Events and Peripheral Artery Disease Burden

**DOI:** 10.3390/jcdd11060157

**Published:** 2024-05-21

**Authors:** Oskari Niiranen, Juha Virtanen, Ville Rantasalo, Amer Ibrahim, Maarit Venermo, Harri Hakovirta

**Affiliations:** 1 Department of Surgery, University of Turku, 20520 Turku, Finland; oskari.e.niiranen@utu.fi (O.N.);; 2 Department of Vascular Surgery, University of Helsinki and Helsinki University Hospital, 00100 Helsinki, Finland; juha.virtanen@hus.fi (J.V.); maarit.venermo@hus.fi (M.V.); 3 Department of Surgery, KFSHRC, King Faisal Specialist Hospital and Research Centre, Madinah 11211, Saudi Arabia; iamer@kfshrc.edu.sa; 4 Department of Surgery, Satasairaala, 28500 Pori, Finland

**Keywords:** MACE, MALE, PAD, heart failure, myocardial infarct, cerebrovascular event, atherosclerosis, tibial artery

## Abstract

Objective: The aim of the present study was to investigate the possible relationship between the segmental burden of lower limb atherosclerosis and Major Adverse Cardiovascular Events (MACEs). Methods: All the consecutive symptomatic peripheral artery disease (PAD) patients admitted for digital subtraction angiography (DSA) at Turku University Hospital department of Vascular Surgery between 1 January 2009 and 30 July 2011 were retrospectively analyzed. Angiography due to symptomatic PAD was used as the index date for the inclusion in the study. The segmental burden of atherosclerosis based on DSA was divided into three categories according to the highest disease burden of the defined artery segment: aorto-iliac, femoropopliteal, or tibial segments. The major association for the study was MACEs (defined as a cerebrovascular event, heart failure (HF) and myocardial infarction requiring hospital admission). Demographic data and MACEs were obtained from the hospital electronic medical records system. Results. The lower limb atherosclerosis burden of tibial vessels was related to an increased probability for HF (OR 3.9; 95%CI 2.4–6.5) and for MACEs overall (OR 2.3; 95%CI 1.4–3.6). The probability of both HF and MACEs overall rose with the increasing severity of the atherosclerosis burden. Moreover, the more severe the tibial vessel atherosclerosis, the higher the risk of HF and MACEs. The most extensive tibial atherosclerosis patients had an OR 4.5; 95%CI 2.6–8.0 for HF and an OR 3.1; and 95%CI 1.7–5.6 for MACEs overall. The femoropopliteal disease burden was also associated with an increased risk of HF (OR 2.3; 95%CI 1.6–3.2) and MACE (OR 1.9; 95%CI 1.3–2.7). However, the increasing extent of atherosclerosis of the femoropopliteal segment solely increased the risk of MACEs. Conclusions: PAD patients with severe tibial atherosclerosis are likely to present with MACEs. The risk is further enhanced as the extent of tibial vessel atherosclerosis is increased. An association between MACE and severe atherosclerosis on the aortoiliac segment was not detected. However, when the femoropopliteal segment was the most affected artery segment, the risk of MACEs was increased.

## 1. Introduction

Peripheral artery disease (PAD) is a major risk factor for a poor cardiovascular outcome. It affects more than 200 million people worldwide [1] and in addition to mortality, it has a significant impact on patients’ daily functioning. There is a strong body of evidence in the present literature that supports the use of non-invasive pressure measurements for predicting the overall and cardiovascular mortality [2,3,4,5,6,7]. Both the non-invasive measures of the ankle brachial index (ABI) and the less utilized toe brachial pressure index (TBI) indirectly reflect the severity of atherosclerotic lesions between the descending aorta and the ankle or toe arteries. The possible significance of the extent of atherosclerosis on the lower limb vessels and patient outcome is poorly understood, however.

Peripheral arteries can be divided into three distinct segments and in each segment, the development of atherosclerosis is associated with distinctive risk factors [8]. For example, smoking, male sex and younger age are associated with aortoiliac lesions [9], whereas male sex and smoking are associated with both internal and external iliac calcification [10]. Both smoking and COPD are risk factors [11] associated with atherosclerosis of the femoropopliteal segment. Diabetes, hypertension, heart failure (HF) and CKD are associated with the tibial and pedal vessel disease [12].

Multiple studies have investigated the clinical significance of atherosclerosis in one or more of these artery segments. Abdominal aortic calcification is associated with cardiovascular mortality, and coronary and cerebrovascular events. The risks for coronary and cerebrovascular events therefore increase along with the extent of abdominal aortic atherosclerosis [13,14]. A retrospective study of infrainguinal atherosclerosis assessed Bollinger scores and demonstrated that the Bollinger score is an independent predictor of mortality. Furthermore, the authors concluded that both the femoropopliteal and tibial Bollinger scores predicted mortality [15]. Recent studies compared the extent of tibial atherosclerosis (crural index (CXi)) and observed an association between the extent of tibial atherosclerosis and several poor outcomes such as cardiovascular mortality, overall mortality amputation free survival, ischemic degenerative brain changes and poor outcome after thrombolysis [16,17,18,19]. However, the cardiovascular outcome in relation to the burden of atherosclerosis on each lower limb artery segment still requires further investigation.

Since the association between extensive atherosclerosis and cardiovascular morbidity is still controversial, the aim of the present study was to investigate whether the extent of aortoiliac, femoropopliteal or tibial disease has an impact on the major adverse cardiovascular events (MACEs) in PAD patients with major adverse limb events (MALEs).

## 2. Materials and Methods

### 2.1. Patient Characteristics

The present study was a retrospective study of 732 symptomatic peripheral artery disease (PAD) patients at Turku University Hospital’s department of vascular surgery admitted for digital subtraction angiography either for diagnostic digital subtraction angiography (DSA) or for endovascular treatment of lower limb atherosclerosis between 1 January 2009 and 30 July 2011. The angiography date was used as the index date for MALEs and an index date for inclusion in the study. Data were collected from hospital electronic databases. The study was approved by the Hospital District of South-Western Finland Ethics Committee (decision ID TK-53-1266-15). Patient consent was not required due to the retrospective nature of the study. This study conformed to the ethics guidelines of the 1975 Declaration of Helsinki.

### 2.2. DSA Analysis and Description of the Crural Index (CXi)

All analyses of segment-specific atherosclerosis were based on DSA images. The index classification was as described in TASC II for aortoiliac and femoropopliteal segments (TASC A–D converted to indices I–IV). All three tibial vessels were analyzed separately for the CXi. Each crural vessel was coded as follows: no detectable occlusive disease or minor stenosis: 0; total occlusion of less than 5 cm: 1; total occlusion of less than 10 cm: 2; total occlusion of less than 15 cm: 3; total occlusion of more than 15 cm: 4. Only total occlusions were measured; other atherosclerotic lesions were not considered. The CXi was created by a sum of the three values that had been obtained from each individual tibial vessel. If the sum was 0, the index was 0, if the sum was between 1 and 3 the index was I, if the sum was 4–6 the index was II, if the sum was 7–9 the index was III and if the sum was 10–12 the index was IV.

Each patient was assigned to a specific group of disease burden (1) aortoiliac, (2) femoropopliteal or (3) crural, based on which 0-IV rating gave the highest number [16,17]. The method is further described in our earlier publications [16,17,20].

### 2.3. Base Line Characteristics and MACEs

The baseline characteristics were collected from the hospital’s patient electronic medical records, retrospectively. Comorbidities were recorded according to ICD-10 codes: coronary artery disease (CAD) (I20.0–I25.9), hypertension (I10.0–I10.9), (I48.0–I48.9), diabetes mellitus (E10.0–E11.9), chronic obstructive pulmonary disease (COPD) (J44.8), hypercholesterolemia (E78.0) and chronic kidney disease (CKD) (N18.1–N18.9). In addition, a smoking history and Rutherford class were recorded at the index date. The last values of the ABI and TBI before the index date for the study were obtained, and the value of the leg with the lower index was used for analyses. All non-invasive pressures were analyzed in a certified Angio laboratory as described earlier by Wickström and colleagues [21].

A MACE was defined as stroke, HF or another acute coronary syndrome. MACE data for the study cohort of 732 patients with a MACE were obtained from hospital digital patient files before and after the study index date.

### 2.4. Statistical Analyses

Statistical analyses were performed using the IBM SPSS^®^ version 29 statistics program. The Shapiro–Wilk test was used to test the normality of the study data. Continuous variables were expressed as mean ± standard deviation (SD) and the Kruskal–Wallis test was used for comparisons. Categorical variables were expressed as frequency and percentage and comparisons were performed using the Chi-square test. Age-adjusted logistic regression analyses were performed on MACE overall data and all selected MACEs separately as the outcome. Significant burdens were selected for the multilogistic regression analyses based on age-adjusted regression analyses. The following confounding variables were added to the model: Age, CAD, Diabetes, CKD. All authors had full access to all the study data. The corresponding author takes the responsibility for the integrity of the data analyses. Data cannot be shared publicly because of patient identification. However, data are available from the corresponding author for researchers who meet the criteria for access to confidential data.

## 3. Results

### 3.1. Demography and MACEs

The patients’ demographic data are presented on Table 1, Table 2, Table 3 and Table 4. Altogether, 489 (66.8% of the cohort) patients presented with one or more MACEs. Myocardial infarct (MI) was the most abundant (*n* = 303 patients; 41.4%), followed by HF (*n* = 268 patients; 36.6%) and cerebrovascular incidents (*n* = 209 patients; 28.6%). Both HF and MI were diagnosed in 160 patients (21.9%), with MI and cerebrovascular incidents in 87 patients (11.9%), and HF and cerebrovascular incidents in 83 patients (11.3%).

### 3.2. The Risk of a MACE and Atherosclerosis Burden

An age-adjusted logistic regression analysis was performed to analyze the possible association between the atherosclerosis burden group and risk of defined outcomes. The aortoiliac or femoropopliteal segment did not present an increased OR (odds ratio) for any MACEs (Table 5 and Table 6). Extensive tibial artery atherosclerosis was associated with MACEs (Table 7). When analyzed against the segment-specific burden of atherosclerosis, the infrainguinal disease femoropopliteal and tibial burden was associated with an increased risk compared to severe aortoiliac segment disease (Table 8).

Based on the age-adjusted regression analyses, the CXi and lower limb artery disease burden was further analyzed via multilogistic regression analyses. The model contained significant comorbidities in regard to CAD, CKD and diabetes; see Table 9.

## 4. Discussion

The present study suggests that extensive tibial vessel atherosclerosis is associated with an increased risk of HF and MACE overall. In addition, both infrainguinal lower limb atherosclerosis burdens, i.e., of the femoropopliteal segment and the tibial segment, are associated with an increased risk of either HF or MACEs in general. In contrast, the aortoiliac burden could not be demonstrated to be associated with any consistent risk of any of the selected outcomes. Our earlier studies on the overall and cardiovascular mortality based on this cohort are in line with the present observations that the highest cardiovascular disease burden is associated with extensive and severe tibial vessel disease [16,17].

The inclusion criteria for this cohort were MALEs. Therefore, every patient in this cohort had significant PAD. The present study did not demonstrate an association between aortoiliac segment disease and MACEs. However, compared to the normal population, the cardiovascular burden of these patients was increased [22,23], and this was further borne out by the logistic regression analyses. The classification utilized for aortoiliac and femoropopliteal segments is based on the TASC A-D criteria. These criteria might not best present the extent of atherosclerosis on the aortoiliac segment of patients for all categories, but rather present the complicity of revascularization in that TASC class. Therefore, AI III and IV might especially comprise patients that had lesions that were technically difficult to treat but who did not have extensive atherosclerosis at that particular segment [24].

Even one in seven CAD or PAD patients present with a new MACE or MALE within 2 years of follow-up [25]. Therefore, these patients have a large economic burden, and targeted efficient secondary preventive actions for patients with the highest MACE risk is therefore essential [25]. Some general health-related predictors for MACEs and MALEs among patients with CAD and/or MALEs have previously been identified [25]. Based on the present observations, the analyses of the segment-specific burden of atherosclerosis among MALE patients enable the identification of those with the highest risk of HF and MACEs overall. In accordance with the present observations, HF has been shown to be associated with low ABI indices [26]. In that study, which was an Atherosclerosis Risk in Communities (ARIC) study, Gupta et al., 2014 also demonstrated a threshold value of 1.0 [26] for the increased risk of MACEs. In addition, borderline ABI values of 0.9–1.0 were shown to be associated with an increased risk of HF. However, the present observations are, to our knowledge, the first observations that suggest that the extent and anatomic distribution of atherosclerosis of the lower limb arteries can have a significant effect on the burden of HF among patients with symptomatic PAD. The mean ABI for patients at the highest risk of HF for CXi category IV was 0.57 and that for the segment-specific tibial vessel atherosclerosis burden was 0.97, which suggests that the ABI alone cannot distinguish patients with the highest risk.

The utilized parameters for disease extent and severity are based on the clinician’s evaluation of DSA images. The categories and grading of atherosclerosis severity based on the TASC II classification was originally created to serve as a guide to select the strategy for revascularization. The CXi is based on the length of the occlusions of three tibial arteries. For example, a patient with the CXi IV disease has extensive severe lesions in all tibial arteries, and mild lesions are not taken into consideration when the index is calculated. All these aspects should be considered when interpreting the present observations. However, the present observations strongly suggest that the localization, extent and severity of atherosclerosis of three lower limb artery segments has a significant input on the patient’s outcome. Therefore, the development of algorithms that can automatically sequence the lower limb arteries and objectively analyze the disease burden [27] is needed. Such algorithms will provide a quick tool for angiography-based risk analyses. The algorithms could predict not only selected MACEs, but also predict cardiovascular death and even overall death.

The present study further emphasizes the need for new means for the risk analyses for peripheral artery disease. The most utilized non-invasive lower limb pressure measurement ABI has many advantages. However, the present results suggest that the ABI together with other parameters such as the extent or severity of the disease burden would further enhance the risk analyses. In the presence of tibial disease, the ABI is especially pseudohypertensive in many patients; thus, it may not distinguish all patients with a significant tibial atherosclerotic burden. Whether the ABI or TBI is more sensitive for risk analyses together with automated artificially intelligent produced data on disease burden will be an interesting topic for further studies as well as the possibility to utilize cytokines and chemokines as biomarkers for the progression and risk of the acute onset of vascular disease in various organ systems [28,29,30].

## 5. Conclusions

According to the present study data, severe tibial vessel PAD is associated with high overall MACE risk and especially the risk of HF. Further studies are essential for creating artificial intelligence-assisted analyses of lower limb atherosclerosis in order to provide a rapid and effective tool for both risk analyses for individual patients and possibly helping the planning of the optimal revascularization for the affected limb.

## Figures and Tables

**Table 1:** The demography and diagnosed MACE conditions of the MALE patients for each aortoiliac (AI) burden group for categorical values n (%) and continuous variables mean (SD).

	AI 0	AI I	AI II	AI III	AI IV	*p* Value
MI	220 (43.1)	26 (30.2)	20 (39.2)	12 (40.0)	25 (45.5)	0.227
HF	204 (40.0)	24 (27.9)	14 (27.5)	10 (33.3)	16 (29.1)	0.073
CeV	149 (29.2)	26 (30.2)	13 (25.5)	4 (13.3)	17 (30.9)	0.392
MACE overall	350 (68.6)	57 (66.3)	30 (58.8)	16 (53.3)	36 (65.5)	0.305
Age	75.4 ± 10.4	74.23 ± 10.8	75.4 ± 10.5	69.3 ± 10.8	73.7 ± 11.0	0.030
Male	291 (57.1)	54 (62.8)	27 (52.9)	19 (63.3)	36 (65.5)	0.552
CAD	219 (42.9)	36 (41.9)	16 (31.4)	14 (46.7)	30 (54.5)	0.198
CeVD	86 (16.9)	17 (19.8)	4 (7.8)	5 (16.7)	11 (20.0)	0.385
Hypertension	359 (70.4)	60 (69.8)	30 (58.8)	18 (60.0)	42 (76.4)	0.245
Diabetes	234 (45.9)	31 (36.0)	14 (27.5)	10 (33.3)	11 (20.0)	<0.001
COPD	52 (10.2)	13 (15.1)	9 (18.0)	11 (36.7)	7 (12.7)	0.002
CKD	57 (11.2)	5 (5.9)	2 (3.9)	2 (6.7)	4 (7.3)	0.320
Hyperlipidaemia	186 (36.5)	31 (36.0)	14 (27.5)	16 (53.3)	26 (47.3)	0.096
Smoking history	112 (22.0)	35 (40.7)	27 (52.9)	15 (50.0)	23 (41.8)	<0.001
Rf 2	10 (2.0)	2 (36.1))	0 (0.0)	0 (0.0)	0 (0.0)	
Rf 3	184 (36.1)	54 (62.8)	39 (76.5)	15 (50.0)	26 (47.3)	
Rf 4	97 (19.9)	17 (19.8)	7 (13.7)	4 (13.3)	15 (27.3)	
Rf 5	105 (20.6)	7 (8.1)	3 (5.9)	4 (13.3)	8 (14.5)	
Rf 6	112 (22.0)	6 (7.0)	2 (3.9)	7 (23.3)	6 (10.9)	<0.001
ABI	0.809 ± 0.671	0.630 ± 0.467	0.670 ± 0.492	0.501 ± 0.205	0.448 ± 0.211	<0.001
TBI	0.294 ± 0.174	0.339 ± 0.180	0.329 ± 0.135	0.309 ± 0.189	0.251 ± 0.170	0.029

ABI: ankle brachial index, AI; aortoiliac, CAD: coronary artery disease, CeV: cerebrovascular event, CeVD: cerebrovascular disease, CKD: chronic kidney dysfunction, COPD: chronic obstructive pulmonary disease, HF: heart failure, MACE: major adverse cardiovascular event, Rf: Rutherford category, TBI; toe brachial index.

**Table 2:** The demography and diagnosed MACE conditions of the MALE patients for each femoropopliteal (FP) burden group for categorical values n (%) and continuous variables mean (SD).

	FP 0	FP I	FP II	FP III	FP IV	*p* Value
MI	63 (36.0)	23 (33.3)	63 (52.1)	41 (43.2)	113 (41.5)	0.046
HF	69 (39.4)	17 (24.6)	46 (38.0)	34 (35.8)	102 (37.5)	0.268
CeV	59 (33.7)	18 (26.1)	37 (30.6)	21 (22.1)	74 (27.2)	0.303
MACE (overall)	118 (67.4)	41 (59.4)	89 (73.6)	64 (67.4)	177 (65.1)	0.329
Age	75.5 ± 11.1	75.4 ± 9.63	76.0 ± 9.54	74.1 ± 10.2	74.2 ± 11.0	0.455
Male	107 (61.1)	42 (60.9)	73 (60.3)	55 (57.9)	150 (55.1)	0.724
CAD	66 (37.7)	24 (34.8)	64 (52.9)	40 (42.1)	121 (44.5)	0.063
CeVD	27 (15.4)	15 (21.7)	20 (16.5)	20 (21.1)	41 (15.1)	0.499
Hypertension	112 (64.0)	52 (75.4)	90 (74.4)	72 (75.8)	183 (67.3)	0.123
Diabetes	71 (40.6)	32 (46.4)	53 (43.8)	44 (46.3)	100 (36.8)	0.354
COPD	13 (7.5)	8 (11.6)	9 (7.4)	15 (16.0)	47 (17.3)	0.008
CKD	29 (16.6)	6 (8.7)	8 (6.6)	9 (9.5)	18 (6.6)	0.012
Hyperlipidaemia	58 (33.1)	28 (40.6)	43 (35.5)	39 (41.1)	105 (38.6)	0.633
Smoking history	44 (25.1)	23 (33.3)	28 (23.1)	33 (34.7)	84 (30.9)	0.202
Rf 2	1 (0.6)	2 (2.9)	6 (5.0)	0 (0.0)	3 (1.1)	
Rf 3	71 (40.6)	40 (58.0)	59 (48.8)	48 (50.5)	100 (36.8)	
Rf 4	25 (14.3)	7 (10.1)	15 (12.4)	19 (20.0)	74 (27.2)	
Rf 5	36 (20.6)	6 (8.7)	18 (14.9)	15 (15.8)	52 (19.1)	
Rf 6	42 (24.0)	14 (20.3)	23 (19.0)	11 (11.6)	43 (15.8)	<0.001
ABI	1.08 ± 0.760	0.779 ± 0.633	0.784 ± 0.582	0.611 ± 0.493	0.532 ± 0.420	<0.001
TBI	0.326 ± 0.166	0.308 ± 0.166	0.353 ± 0.190	0.299 ± 0.180	0.255 ± 0.161	<0.001

ABI: ankle brachial index, CAD: coronary artery disease, CeV: cerebrovascular event, CeVD: cerebrovascular disease, CKD: chronic kidney dysfunction, COPD: chronic obstructive pulmonary disease, FP: femoropopliteal, HF: heart failure, MACE: major adverse cardiovascular event, Rf: Rutherford category, TBI: toe brachial index.

**Table 3:** The demography and diagnosed MACE conditions of the MALE patients at each tibial crural index (CXi) burden group for categorical values n (%) and continuous variables mean (SD).

	CXi O	CXi I	CXi II	Cxi III	Cxi IV	*p* Value
MI	43 (36.8)	26 (41.3)	78 (37.5)	103 (45.8)	53 (44.5)	0.332
HF	22 (18.8)	16 (25.4)	69 (33.2)	100 (44.4)	61 (51.3)	<0.001
CeV	31 (26.5)	17 (27.0)	57 (27.4)	61 (27.1)	43 (36.1)	0.419
MACE overall	66 (56.4)	40 (63.5)	132 (63.5)	156 (69.3)	95 (79.8)	0.002
Age	70.9 ± 9.16	72.4 ± 10.5	75.5 ± 9.82	75.3 ± 10.6	78.3 ± 11.6	<0.001
Male	67 (57.3)	47 (74.6)	132 (63.5)	116 (51.6)	65 (54.6)	0.006
CAD	49 (41.9)	23 (36.5)	83 (39.9)	107 (47.6)	53 (44.5)	0.414
CeVD	17 (14.5)	9 (14.3)	37 (17.8)	42 (18.7)	18 (15.1)	0.828
Hypertension	77 (65.8)	42 (66.7)	140 (67.3)	162 (72.0)	88 (73.9)	0.513
Diabetes	37 (31.6)	30 (47.6)	86 (41.3)	97 (43.1)	50 (42.0)	0.203
COPD	19 (16.2)	7 (11.1)	36 (17.4)	21 (9.4)	9 (7.6)	0.032
CKD	6 (5.1)	9 (14.3)	21 (10.1)	30 (13.3)	4 (3.4)	0.006
Hyperlipidaemia	55 (47.0)	28 (44.4)	73 (35.1)	86 (38.2)	31 (26.1)	0.010
Smoking history	66 (56.4)	25 (39.7)	71 (34.1)	36 (16.0)	14 (11.8)	<0.001
Rf 2	3 (2.6)	1 (1.6)	4 (1.9)	0 (0.0)	4 (3.4)	
Rf 3	77 (65.8)	40 (63.5)	94 (45.2)	84 (37.3)	4 (3.4)	
Rf 4	18 (15.4)	7 (11.1)	41 (19.7)	47 (20.9)	27 (22.7)	
Rf 5	13 (11.1)	5 (7.9)	17 (8.2)	51 (22.7)	41 (34.5)	
Rf 6	5 (4.3)	9 (14.3)	52 (25.0)	43 (19.1)	24 (20.2)	<0.001
ABI	0.643 ± 0.397	0.898 ± 0.643	0.774 ± 0.632	0.811 ± 0.704	0.570 ± 0.482	<0.001
TBI	0.335 ± 0.178	0.386 ± 0.156	0.321 ± 0.163	0.273 ± 0.174	0.222 ± 0.159	<0.001

ABI: ankle brachial index, CAD: coronary artery disease, CeV: cerebrovascular event, CeVD: cerebrovascular disease, CKD: chronic kidney dysfunction, COPD: chronic obstructive pulmonary disease, CXi: crural index, HF: heart failure, MACE: major adverse cardiovascular event, Rf: Rutherford category, TBI: toe brachial index.

**Table 4:** The demography and diagnosed MACE conditions the MALE patients according to the lower limb segment specific atherosclerosis burden groups for categorical values n (%) and continuous variables mean (SD).

	AI	FP	Tibial	*p* Value
MI	50 (38.8)	155 (42.2)	98 (42.1)	0.779
HF	28 (21.7)	118 (32.2)	121 (51.9)	<0.001
CeV	35 (27.1)	89 (24.3)	82 (35.2)	0.015
MACE overall	76 (58.9)	232 (63.2)	178 (76.4)	<0.001
Age	72.8 ± 10.4	74.4 ± 10.0	76.8 ± 11.2	0.001
Male	87 (67.4)	152 (41.4)	109 (46.8)	0.031
CAD	55 (42.6)	161 (43.9)	99 (42.5)	0.939
CeVD	20 (15.5)	367 (17.4)	38 (16.3)	0.879
Hypertension	88 (68.2)	249 (67.8)	169 (72.5)	0.456
Diabetes	30 (23.3)	144 (39.2)	124 (53.2)	<0.001
COPD	23 (17.8)	57 (15.6)	11 (4.7)	<0.001
CKD	8 (6.2)	30 (8.2)	30 (12.9)	<0.001
Hyperlipidaemia	52 (40.3)	152 (41.4)	69 (29.6)	0.010
Smoking history	68 (52.7)	121 (33.0)	23 (9.9)	<0.001
Rf 2	1 (0.8)	7 (1.9)	4 (1.7)	
Rf 3	87 (67.4)	183 (49.9)	48 (20.6)	
Rf 4	23 (17.8)	79 (21.5)	36 (15.5)	
Rf 5	11 (8.5)	41 (11.2)	74 (31.8)	
Rf 6	7 (5.4)	55 (15.0)	71 (30.5)	<0.001
ABI	0.557 ± 0.260	0.650 ± 0.260	0.971 ± 0.782	<0.001
TBI	0.324 ± 0.173	0.308 ± 0.177	0.270 ± 0.165	0.008

ABI: ankle brachial index, AI: aortoiliac, CAD: coronary artery disease, CeV: cerebrovascular event, CeVD: cerebrovascular disease, CKD: chronic kidney dysfunction, COPD: chronic obstructive pulmonary disease, FP: femoropopliteal, HF: heart failure, MACE: major adverse cardiovascular event, Rf: Rutherford category, TBI: toe brachial index.

**Table 5:** The age-adjusted hazard for the overall MACE and MACEs (heart failure, myocardial infarct, cerebrovascular event) in five aortoiliac (AI) burden groups (an OR with 95% confidence intervals and *p* values).

	**HF**		
	**OR**	**95% CI**	***p* Value**
AI 0	Reference		
AI I	0.581	0.345–0.949	0.034
AI II	0.568	0.290–1.05	0.083
AI III	0.750	0.331–1.60	0.469
AI IV	0.615	0.326–1.11	0.118
	**MI**		
	**OR**	**95% CI**	***p* Value**
AI 0	Reference		
AI I	0.571	0.344–0.925	0.026
AI II	0.850	0.466–1.52	0.590
AI III	0.879	0.405–1.85	0.736
AI IV	1.098	0.624–1.92	0.742
	**CeV**		
	**OR**	**95% CI**	***p* Value**
AI 0	Reference		
AI I	0.952	0.584–1.59	0.848
AI II	1.206	0.640–2.41	0.576
AI III	2.683	1.02–9.21	0.071
AI IV	0.923	0.513–1.72	0.793
	**MACE**		
	**OR**	**95% CI**	***p* Value**
AI 0	Reference		
AI I	0.899	0.558–1.47	0.665
AI II	0.653	0.364–1.19	0.156
AI III	0.522	0.249–1.11	0.086
AI IV	0.866	0.487–1.58	0.631

AI: aortoiliac, CeV: cerebrovascular event, 95% CI: 95% confidence interval; HF: heart failure, MACE: major adverse cardiovascular event, MI: myocardial infarct, OR: odds ratio.

**Table 6:** The age-adjusted hazards for the overall MACE and MACEs (heart failure, myocardial infarct, cerebrovascular event) in the defined femoropopliteal (FP) burden groups are presented with 95% confidence intervals and *p* values.

	**HF**		
	**OR**	**95% CI**	***p* Value**
FP 0	Reference		
FP I	0.502	0.263–0.924	0.031
FP II	0.942	0.584–1.52	0.806
FP III	0.856	0.508–1.43	0.557
FP IV	0.922	0.624–1.36	0.682
	**MI**		
	**OR**	**95% CI**	***p* Value**
FP 0	Reference		
FP I	0.889	0.488–1.59	0.695
FP II	1.931	1.21–3.10	0.006
FP III	1.350	0.809–2.25	0.249
FP IV	1.263	0.855–1.87	0.242
	**CeV**		
	**OR**	**95% CI**	***p* Value**
FP 0	Reference		
FP I	1.441	0.784–2.74	0.250
FP II	1.155	0.704–1.91	0.571
FP III	1.792	1.02–3.24	0.048
FP IV	1.361	0.900–2.05	0.142
	**MACE**		
	**OR**	**95% CI**	***p* Value**
FP 0	Reference		
FP I	0.707	0.399–1.26	0.238
FP II	1.343	0.808–2.26	0.259
FP III	0.997	0.587–1.71	0.992
FP IV	0.900	0.600–1.34	0.608

CeV: cerebrovascular event, 95% CI: 95% confidence interval; FP: femoropopliteal, HF: heart failure, MACE: major adverse cardiovascular event, MI: myocardial infarct, OR: odds ratio.

**Table 7:** The age-adjusted hazards for the overall MACE and MACEs (heart failure, acute cardiac syndrome, cerebrovascular event) for the defined tibial burden groups (CXi) are presented with 95% confidence intervals and *p* values.

	**HF**		
	**OR**	**95% CI**	***p* Value**
CXi 0	Reference		
CXi I	3.050	0.699–1.47	0.303
CXi II	3.765	1.26–2.14	0.006
CXi III	5.999	2.06–3.46	<0.001
CXi IV	8.298	2.56–4.54	<0.001
	**MI**		
	**OR**	**95% CI**	***p* Value**
CXi 0	Reference		
CXi I	1.209	0.643–2.26	0.552
CXi II	1.033	0.647–1.66	0.894
CXi III	1.453	0.922–2.31	0.110
CXi IV	1.382	0.821–2.34	0.224
	**CeV**		
	**OR**	**95% CI**	***p* Value**
CXi 0	Reference		
CXi I	0.975	0.492–198	0.944
CXi II	0.955	0.568–1.59	0.860
CXi III	0.969	0.580–1.60	0.903
CXi IV	0.637	0.364–1.11	0.112
	**MACE**		
	**OR**	**95% CI**	***p* Value**
CXi 0	Reference		
CXi I	1.344	0.720–2.55	0.358
CXi II	1.342	0.845–2.13	0.212
CXi III	1.747	1.10–2.78	0.018
CXi IV	3.059	1.73–5.52	<0.001

CeV: cerebrovascular event, 95% CI: 95% confidence interval; CXi: crural index, HF: heart failure, MACE: major adverse cardiovascular event, MI: myocardial infarct, OR: odds ratio.

**Table 8:** The age-adjusted hazards for the overall MACE and MACEs (heart failure, acute cardiac syndrome, cerebrovascular event) in the most severely affected lower limb segment burden groups are presented with 95% confidence intervals and *p* values.

	**HF**		
	**OR**	**95% CI**	***p* Value**
AI	Reference		
FP	2.780	1.08–1.71	0.026
Tibial	6.455	2.41–3.90	<0.001
	**MI**		
	**OR**	**95% CI**	***p* Value**
AI	Reference		
FP	1.155	0.768–1.75	0.491
Tibial	1.147	0.740–1.79	0.541
	**CeV**		
	**OR**	**95% CI**	***p* Value**
AI	Reference		
FP	1.163	0.731–1.82	0.516
Tibial	0.686	0.424–0.109	0.117
	**MACE**		
	**OR**	**95% CI**	***p* Value**
AI	Reference		
FP	1.198	0.793–1.80	0.387
Tibial	2.257	1.42–3.59	<0.001

AI: aortoiliac, CeV: cerebrovascular, 95% CI: 95% confidence interval, FP: femoropopliteal, HF: heart failure, MACE: major adverse cardiovascular event, MI: myocardial infarct, OR: odds ratio.

**Table 9:** The multilogistic regression analyses. The most severely affected lower limb segment and CXi are presented with 95% confidence intervals and *p* values. Model with significant comorbidities in regard to CAD, CKD and diabetes.

	**HF**		
	**OR**	**95% CI**	***p* Value**
AI	Reference		
FP	2.280	1.63–3.20	<0.001
Tibial	3.897	2.41–6.46	<0.001
	**MACE**		
	**OR**	**95% CI**	***p* Value**
AI	Reference		
FP	1.883	1.31–2.74	<0.001
Tibial	2.257	1.42–3.59	<0.001
	**HF**		
	**OR**	**95% CI**	***p* Value**
CXi 0	Reference		
CXi I	1.315	0.842–2.06	0.229
CXi II	2.119	1.34–3.37	0.001
CXi III	3.089	1.60–6.18	<0.001
CXi IV	4.542	2.56–8.30	<0.001
	**MACE**		
	**OR**	**95% CI**	***p* Value**
CXi 0	Reference		
CXi I	1.751	1.04–3.02	0.038
CXi II	2.279	1.36–3.93	0.002
CXi III	2.276	1.15–4.52	0.018
CXi IV	3.059	1.73–5.62	<0.001

AI: aortoiliac, 95% CI: 95% confidence interval; FP: femoropopliteal, HF: heart failure, MACE: major adverse cardiovascular event, OR: odds ratio.

## Data Availability

The data are unavailable due patient identification privacy as decided by the Ethics Committee. However, anonymized data can be accessed on demand by contacting the Research Center VARHA (Varsinais-Suomen hyvinvointialue).

## References

[1] Fowkes F.G.R., Rudan D., Rudan I., Aboyans V., Denenberg J.O., McDermott M.M., Norman P.E., Sampson U.K.A., Williams L.J., Mensah G.A. (2013). Comparison of global estimates of prevalence and risk factors for peripheral artery disease in 2000 and 2010: A systematic review and analysis. Lancet.

[2] Hyun S., Forbang N.I., Allison M.A., Denenberg J.O., Criqui M.H., Ix J.H. (2014). Ankle-brachial index, toe-brachial index, and cardiovascular mortality in persons with and without diabetes mellitus. J. Vasc. Surg..

[3] Vogt M.T., McKenna M., Wolfson S.K., Kuller L.H. (1993). The relationship between ankle brachial index, other atherosclerotic disease, diabetes, smoking and mortality in older men and women. Atherosclerosis.

[4] Samba H., Guerchet M., Ndamba-Bandzouzi B., Kehoua G., Mbelesso P., Desormais I., Aboyans V., Preux P.-M., Lacroix P. (2019). Ankle Brachial Index (ABI) predicts 2-year mortality risk among older adults in the Republic of Congo: The EPIDEMCA-FU study. Atherosclerosis.

[5] Criqui M.H., McClelland R.L., McDermott M.M., Allison M.A., Blumenthal R.S., Aboyans V., Ix J.H., Burke G.L., Liu K., Shea S. (2010). The Ankle-Brachial Index and Incident Cardiovascular Events in the MESA (Multi-Ethnic Study of Atherosclerosis). J. Am. Coll. Cardiol..

[6] Aboyans V., Lacroix P., Postil A., Guilloux J., Rollé F., Cornu E., Laskar M. (2005). Subclinical Peripheral Arterial Disease and Incompressible Ankle Arteries Are Both Long-Term Prognostic Factors in Patients Undergoing Coronary Artery Bypass Grafting. J. Am. Coll. Cardiol..

[7] Aboyans V., Criqui M.H., Abraham P., Allison M.A., Creager M.A., Diehm C., Fowkes F.G.R., Hiatt W.R., Jönsson B., Lacroix P. (2012). Measurement and interpretation of the ankle-brachial index: A scientific statement from the American Heart Association. Circulation.

[8] Diehm N., Shang A., Silvestro A., Do D.-D., Dick F., Schmidli J., Mahler F., Baumgartner I. (2006). Association of Cardiovascular Risk Factors with Pattern of Lower Limb Atherosclerosis in 2659 Patients Undergoing Angioplasty. Eur. J. Vasc. Endovasc. Surg..

[9] Chen Q., Smith C.Y., Bailey K.R., Wennberg P.W., Kullo I.J. (2013). Disease Location Is Associated With Survival in Patients With Peripheral Arterial Disease. J. Am. Hear. Assoc..

[10] Hendriks E.J., Beulens J.W., de Jong P.A., van der Schouw Y.T., Sun W.-N., Wright C.M., Criqui M.H., Allison M.A., Ix J.H. (2017). Calcification of the splenic, iliac, and breast arteries and risk of all-cause and cardiovascular mortality. Atherosclerosis.

[11] Gray B.H., Grant A.A., Kalbaugh C.A., Blackhurst D.W., Langan E.M., Taylor S.A., Cull D.L. (2010). The impact of isolated tibial disease on outcomes in the critical limb ischemic population. Ann. Vasc. Surg..

[12] Aboyans V., Desormais I., Lacroix P., Salazar J., Criqui M.H., Laskar M. (2010). The General Prognosis of Patients With Peripheral Arterial Disease Differs According to the Disease Localization. J. Am. Coll. Cardiol..

[13] Bastos Goncalves F., Voute M.T., Hoeks S.E., Chonchol M.B., Boersma E.E., Stolker R.J., Verhagen H.J.M. (2012). Calcification of the abdominal aorta as an independent predictor of cardiovascular events: A meta-analysis. Heart.

[14] Rantasalo V., Laukka D., Nikulainen V., Jalkanen J., Gunn J., Hakovirta H. (2022). Aortic calcification index predicts mortality and cardiovascular events in operatively treated patients with peripheral artery disease: A prospective PURE ASO cohort follow-up study. J. Vasc. Surg..

[15] Tern P.J., Kujawiak I., Saha P., Berrett T.B., Chowdhury M.M., Coughlin P.A. (2018). Site and Burden of Lower Limb Atherosclerosis Predicts Long-term Mortality in a Cohort of Patients With Peripheral Arterial Disease. Eur. J. Vasc. Endovasc. Surg..

[16] Wickström J.-E., Jalkanen J.M., Venermo M., Hakovirta H.H. (2017). Crural Index and extensive atherosclerosis of crural vessels are associated with long-term cardiovascular mortality in patients with symptomatic peripheral artery disease. Atherosclerosis.

[17] Jalkanen J.M., Wickström J.-E., Venermo M., Hakovirta H.H. (2016). The extent of atherosclerotic lesions in crural arteries predicts survival of patients with lower limb peripheral artery disease: A new classification of crural atherosclerosis. Atherosclerosis.

[18] Virtanen J., Varpela M., Biancari F., Jalkanen J., Hakovirta H. (2020). Association between anatomical distribution of symptomatic peripheral artery disease and cerebrovascular disease. Vascular.

[19] Vakhitov D., Hakovirta H., Saarinen E., Oksala N., Suominen V. (2020). Prognostic risk factors for recurrent acute lower limb ischemia in patients treated with intra-arterial thrombolysis. J. Vasc. Surg..

[20] Koivunen V., Juonala M., Venermo M., Laivuori M., Jalkanen J.M., Hakovirta H.H. (2021). Toe pressure and toe brachial index are predictive of cardiovascular mortality regardless of the most diseased arterial segment in symptomatic lower-extremity artery disease—A retrospective cohort study. PLoS ONE.

[21] Wickström J.-E., Laivuori M., Aro E., Sund R., Hautero O., Venermo M., Jalkanen J., Hakovirta H. (2017). Toe Pressure and Toe Brachial Index are Predictive of Cardiovascular Mortality, Overall Mortality, and Amputation Free Survival in Patients with Peripheral Artery Disease. Eur. J. Vasc. Endovasc. Surg..

[22] Criqui M.H., Langer R.D., Fronek A., Feigelson H.S., Klauber M.R., McCann T.J., Browner D. (1992). Mortality over a Period of 10 Years in Patients with Peripheral Arterial Disease. N. Engl. J. Med..

[23] Steg P.G., Bhatt D.L., Wilson P.W.F., D’agostino R., Ohman E.M., Röther J., Liau C.-S., Hirsch A.T., Mas J.-L., Ikeda Y. (2007). One-Year Cardiovascular Event Rates in Outpatients with Atherothrombosis. JAMA.

[24] Norgren L., Hiatt W.R., Dormandy J.A., Nehler M.R., Harris K.A., Fowkes F.G. (2007). Inter-Society Consensus for the Management of Peripheral Arterial Disease (TASC II). Eur. J. Vasc Endovasc. Surg..

[25] Berger A., Simpson A., Bhagnani T., Leeper N.J., Murphy B., Nordstrom B., Ting W., Zhao Q., Berger J.S. (2019). Incidence and Cost of Major Adverse Cardiovascular Events and Major Adverse Limb Events in Patients with Chronic Coronary Artery Disease or Peripheral Artery Disease. Am. J. Cardiol..

[26] Gupta D.K., Skali H., Claggett B., Kasabov R., Cheng S., Shah A.M., Loehr L.R., Heiss G., Nambi V., Aguilar D. (2014). Heart failure risk across the spectrum of ankle-brachial index: The ARIC study (Atherosclerosis Risk In Communities). JACC Heart Fail..

[27] Halkoaho J., Niiranen O., Salli E., Kaseva T., Savolainen S., Kangasniemi M., Hakovirta H. (2024). Quantifying the calcification of abdominal aorta and major side branches with deep learning. Clin. Radiol..

[28] Quagliariello V., Passariello M., Rea D., Barbieri A., Iovine M., Bonelli A., Caronna A., Botti G., De Lorenzo C., Maurea N. (2020). Evidences of CTLA-4 and PD-1 Blocking Agents-Induced Cardiotoxicity in Cellular and Preclinical Models. J. Pers. Med..

[29] Quagliariello V., Bisceglia I., Berretta M., Iovine M., Canale M.L., Maurea C., Giordano V., Paccone A., Inno A., Maurea N. (2023). PCSK9 Inhibitors in Cancer Patients Treated with Immune-Checkpoint Inhibitors to Reduce Cardiovascular Events: New Frontiers in Cardioncology. Cancers.

[30] Jalkanen J., Maksimow M., Hollmén M., Jalkanen S., Hakovirta H. (2016). Compared to Intermittant Claudication Critical Limb Ischemia Is Associated with Elevated Levels of Cytokines. PLoS ONE.

